# Robot-assisted radical prostatectomy in the treatment of patients with clinically high-risk localized and locally advanced prostate cancer: single surgeons functional and oncologic outcomes

**DOI:** 10.1186/s12894-022-00998-6

**Published:** 2022-04-04

**Authors:** Tae Young Shin, Yong Seong Lee

**Affiliations:** 1 Department of Urology, Ewha Womans University Mokdong Hospital, Ewha Womans University College of Medicine, Seoul, 07985 Republic of Korea; 2 Department of Urology, Hallym University Sacred Heart Hospital, Hallym University College of Medicine, Anyang, 14068 Republic of Korea; 3 Department of Urology, Chung-Ang University Gwangmyeong Hospital, Chung-Ang University College of Medicine, Gwangmyeong, 14353 Republic of Korea

**Keywords:** Prostatectomy, Prostate cancer, Robot-assisted surgery, Positive surgical margins, Recurrence

## Abstract

**Background:**

Optimal treatment approaches for high-risk localized and locally advanced prostate cancer remain controversial and there are currently no standard treatments. These patients with high-risk localized and locally advanced prostate cancer are usually offered radiotherapy in combination with hormonal therapy. We report functional and oncologic outcomes of patients who underwent primary robot-assisted radical prostatectomy (RARP) and assess the role of RARP in patients with high-risk localized and locally advanced prostate cancer.

**Methods:**

This study included 188 patients with high-risk localized (clinical stage T2c or a pretreatment prostate-specific antigen level > 20 ng/mL or a biopsy Gleason score ≥ 8) and/or locally advanced (any PSA, cT3-4 or cN+) prostate cancer who underwent RARP between July 2013 and May 2020. Functional outcomes including postoperative continence and potency were assessed at 1, 3, 6, and 12 months after RARP. Oncologic outcomes comprised positive surgical margins (PSMs), biochemical recurrence (BCR), BCR-free survival, and clinical recurrence (CR)-free survival rates at 1 and 3 years.

**Results:**

The median operative time was 185 (interquartile range [IQR] 130–260) minutes. Based on postoperative pathology, the rates of PSMs in the entire cohort and in those with stage pT2 disease were 26.6% and 8.5%, respectively. The continence and potency rates at 12 months were 88.3% and 56.4%, respectively. The BCR rate was 22.3%, and the median time to BCR was 10.5 (IQR 3.5–26.9) months. The 1- and 3-year BCR-free survival rates were 87.6% and 78.7%, respectively, and the 1- and 3-year CR-free survival rates were 97.5% and 90.8%, respectively.

**Conclusions:**

Most patients with clinically high-risk localized and locally advanced prostate cancer treated with primary RARP remained BCR-free and CR-free during the 1- and 3-year follow-up, demonstrating the good functional outcomes with RARP. RARP was a safe and feasible minimally invasive surgical alternative to radiotherapy or hormonal therapy in select patients with high-risk localized and locally advanced prostate cancer. These results should be validated to assure the reproducibility of measurements in prospective randomized-controlled studies on primary RARP.

## Background

Prostate cancer is the most common non-cutaneous malignancy in males in the USA and the second leading cause of cancer-related deaths in males [1, 2]. Prostate cancer occurs globally, with a rapidly increasing incidence in Asia, and many etiological factors are being studied [3]. Following the implementation of early screening and prostate biopsy, most patients are diagnosed with organ-confined prostate cancer, which is potentially curable [4]. In patients with localized prostate cancer, robot-assisted radical prostatectomy (RARP) is a standard surgical treatment besides open radical prostatectomy [5]. However, 20–30% of patients with prostate cancer at initial diagnosis harbor clinically high-risk disease with localized and locally advanced pathology, defined as D'Amico high-risk classification and according to the EAU-ESTRO-SIOG guidelines [6–8]. Due to the underlying aggressive pathology, patients with high-grade prostate cancer and a Gleason score 8–10 may subsequently experience disease recurrence, which may result in early metastasis with significant morbidity and eventual mortality; up to 85% of these patients die of prostate cancer within 10 years of diagnosis [9]. Therefore, optimal treatment approaches for high-risk localized and locally advanced prostate cancer remain controversial and there are currently no standard treatments. Many surgeons are reluctant to perform RARP in patients with clinically high-risk localized and locally advanced prostate cancer, given the relative advantage of the procedure [10]. Specifically, worse oncologic and functional outcomes are anticipated after radical prostatectomy in patients with high-risk localized and locally advanced prostate cancer. Thus, these patients usually offered radiotherapy in combination with hormonal therapy, which is however an incomplete therapeutic approach with significant side effects [11, 12].

Recently, several studies have described reasonable oncologic outcomes and survival advantages with radical prostatectomy as first-line therapy in patients with high-risk prostate cancer [13, 14]. RARP represents a well-standardized, safe, and oncological effective option in patients with locally advanced prostate cancer [15]. Thus, the European Urology Association guidelines state that radical prostatectomy can be offered as a first-line therapy in patients with high-risk prostate cancer as part of multimodality treatment [16].

The objective of the present study was to evaluate functional and oncologic outcomes at 1 and 3 years after surgery in patients with clinically high-risk localized and locally advanced prostate cancer who underwent primary RARP.

## Methods

### Study population

This study was a retrospective, non-randomized study. Medical records of high-risk 780 patients who underwent RARP by a single experienced surgeon (> 1000 RARPs) were retrospectively reviewed between July 2013 and May 2020 at our institution. Inclusion and exclusion criteria were: (1) we included 208 patients with 1-year of follow-up and those who had preoperative high-risk localized and locally advanced prostate cancer, (2) exclusion criteria were any neoadjuvant hormonal treatment, prior radiation therapy, and previous history of urethral stricture and urinary incontinence, (3) we excluded 14 patients who presented insufficient data and 6 patients who were transferred to our institution after being diagnosed with prostate cancer in other hospitals. Finally, 188 of the 780 patients were included in the study. All patients underwent preoperative multiparametric magnetic resonance imaging (mpMRI) to determine clinical stage using the location and distribution of prostate cancer. High-risk localized prostate cancer (28.7%, 54 of the 188 patients) was defined using the D'Amico classification [7]. High-risk locally advanced prostate cancer (72.3%, 134 of the 188 patients) was defined using the EAU-ESTRO-SIOG guidelines [8]. The details of the surgical procedure used in these patients were previously published [17]. Pelvic lymph node dissection (PLND) was performed in all patients. Extended PLND (ePLND) until common iliac artery area was performed in 170 patients (90.4%) with a risk of lymph node involvement of > 5% in the Briganti nomogram [18]. None of the patients had received neoadjuvant hormonal therapy before RARP.

### Study design

The study was approved by the Hallym University Sacred Heart Hospital Ethics Committee (approval No. 2018-05-012). Written informed consent was obtained from all patients. Data were collected in a customized database and analyzed. All methods were performed in accordance with relevant institutional guidelines and regulations. We assessed the following demographic data: age, body mass index, American Society of Anesthesiologists score, prostate volume, PSA level, and biopsy Gleason score. Comorbidities were assessed using the age-adjusted Charlson comorbidity index scoring system [19]. Baseline sexual function before RARP was assessed using the Sexual Health Inventory for Men (SHIM) questionnaire, and preoperative urinary function was evaluated using the International Prostate Symptom Score (IPSS). Postoperative complications were recorded and evaluated using the Clavien–Dindo classification [20]. Postoperatively, there are routinely follow-up schedules of asymptomatic patients by obtaining at least a disease-specific history and serum PSA measurement. These should be performed at 3, 6, 9 and 12 months after treatment, then every 6 months until 3 years, and then annually.

The primary endpoint was postoperative functional at 1–12 months and oncologic outcomes at 1–3 years after RARP. At 1, 3, 6, and 12 months after RARP, we evaluated potency rates using the SHIM questionnaire and continence rates using a daily pad-weighing test with the IPSS for urinary function. Postoperative return of erectile function was scored as ≥ 4 on question 2 of the SHIM questionnaire or the ability to have successful sexual intercourse. Patients with zero pad use per day were considered as postoperative recovery of continence. The following pathologic variables after RARP were also evaluated: pathologic stage, Gleason score, and positive surgical margins (PSMs). Oncologic outcomes comprised biochemical recurrence (BCR), BCR-free survival, and clinical recurrence (CR)-free survival rates at one and three years after RARP. According to AUA guidelines, BCR was defined as two consecutive PSA values ≥ 0.2 ng/mL [21]. CR was defined as local recurrence and/or distant metastasis confirmed by histology and/or imaging. Salvage therapy was defined as the implementation of radiotherapy or hormonal therapy more than 6 months after RARP and/or in the presence of BCR, or radiotherapy or hormonal therapy delivered within 6 months after RARP with a detectable PSA value (≥ 0.1 ng/mL) when radiotherapy or hormonal therapy was administered, following an earlier undetectable PSA value [22]. Patients who underwent salvage therapy were categorized to have developed BCR. Adjuvant therapy was defined as the implementation of radiotherapy or hormonal therapy within six months following RARP in the absence of BCR.

### Statistical analysis

Continuous variables were reported as medians with interquartile ranges (IQRs) and categorical variables were reported as percentages. Continence, potency, BCR-free survival, and CR-free survival after RARP were estimated using Kaplan–Meier analysis. All statistical analyses were performed using SPSS Statistics for Windows version 26.0 (IBM, Armonk, NY, USA).

## Results

The baseline demographic, clinical, and pathologic data of the 188 patients included in the study are summarized in Table 1. The median operative time was 185 (IQR 130–260) minutes, the estimated blood loss was 200 (IQR 150–450) mL, and no patient experienced intraoperative complications. Pelvic lymphadenopathy was present in 12 of the 188 patients (6.4%). The catheter was removed 1 week after surgery in all patients. The overall postoperative Clavien–Dindo grade I–II complications were developed in 22 patients (11.7%). Within the first year after RARP, five patients (2.7%) developed Clavien–Dindo grade ≥ III complications such as lymphocele and urinary retention, which would require additional intervention.

**Table 1:** Preoperative characteristics of 188 patients with clinically high-risk localized and locally advanced prostate cancer

Parameters	N = 188
Age, median (IQR), year	62.5 (51.0–79.0)
BMI, median (IQR), kg/m^2^	25.4 (23.6–29.5)
ASA score, median (IQR)	2.0 (1.0–2.0)
PSA, median (IQR), ng/ml	28.85 (11.5–150.8)
Prostate volume, median (IQR), cc	38.6 (23.5–110.0)
Biopsy Gleason score	
6	4 (2.1%)
7	58 (30.9%)
8	82 (43.6%)
≥ 9	44 (23.4%)
Clinical stage	
High-risk localized (≤ cT2c)	54 (28.7%)
High-risk locally advanced	134 (71.3%)
cT3a	72 (38.3%)
≥ cT3b	62 (33.0%)
cN1	10 (5.3%)
SHIM score, median (IQR)	16.5 (7–24)
IPSS score, median (IQR)	14.5 (4–28)
Charlson comorbidity index	
0	148 (78.7%)
1–2	35 (18.6%)
≥ 3	5 (2.7%)

**Fig. 1:**
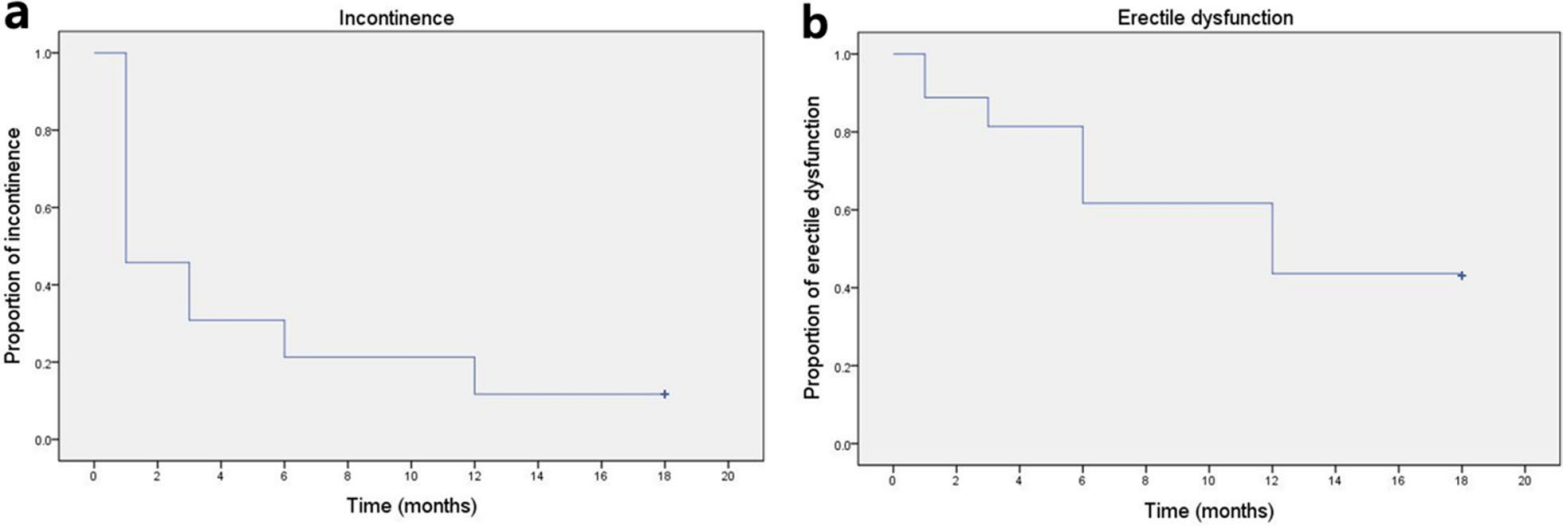
Kaplan–Meier curve showing estimated postoperative incontinence (**a**) and erectile dysfunction (**b**) of 188 patients with clinically high-risk localized and locally advanced prostate cancer

The continence rates were 54.3%, 69.2%, 78.7%, and 88.3% and the potency rates were 11.2%, 18.6%, 38.3%, and 56.4% at 1, 3, 6, and 12 months after RARP, respectively (Table 2). Figure 1 shows the Kaplan–Meier curve for incontinence (a) and erectile dysfunction (b). Most of the patients received penile rehabilitation with regular use of oral phosphodiesterase type 5 inhibitors, starting one week after RARP until recovery of erectile function. The median times to continence and potency recovery were 2.3 (IQR 0.5–12.5) and 8.5 (IQR 3.2–15.5) months, respectively.

**Table 2:** Data on continence and potency recovery during 1-year follow-up after RARP

Time	Patients achieving continence, N (%)	Patients achieving potency, N (%)
1 month	102 (54.3%)	21 (11.2%)
3 months	130 (69.2%)	35 (18.6%)
6 months	148 (78.7%)	72 (38.3%)
12 months	166 (88.3%)	106 (56.4%)

Table 3 shows the histopathologic findings and follow-up data. Briefly, 63 of the 188 patients (33.5%) presented with stage pT2 cancer indicating organ-confined disease based on final pathological examination. Among the remaining 125 patients (66.5%) with stage pT3 cancer, extraprostatic extension (stage pT3a) and seminal vesicle invasion (pT3b) were found in 75 (39.9%) and 50 (26.6%) patients, respectively. Additionally, 81 (43.1%), 74 (39.4%), and 33 (17.6%) patients had postoperative Gleason scores of 7, 8, and ≥ 9, respectively. In the present study, 126 patients (67.0%) had a preoperative biopsy Gleason score of ≥ 8 whereas 107 patients (56.9%) had a postoperative pathologic Gleason score of ≥ 8. Therefore, pathologic downgrading at the time of final pathologic examination after RARP was observed in 10.1% of the patients with high-risk localized and locally advanced prostate cancer. Finally, 100 of the 188 patients (53.2%) did not need secondary therapy such as radiotherapy or hormonal therapy during the 3-year follow-up period.

**Table 3:** Intraoperative, histopathologic, and postoperative data of 188 patients with high-risk localized and locally advanced prostate cancer undergoing RARP

Parameters	N = 188
Operative time, median (IQR), minutes	185 (130–260)
Blood loss, median (IQR), ml	200 (150–450)
Blood transfusion	1 (0.5%)
PLND	
Extended PLND	170 (90.4%)
Limited PLND	18 (9.6%)
Nodal involvement	12 (6.4%)
Complications	
Clavien grade I, II	22 (11.7%)
Clavien grade ≥ III	5 (2.7%)
Pathologic stage	
High-risk localized (≤ pT2c)	63 (33.5%)
High-risk locally advanced	125 (66.5%)
pT3a	75 (39.9%)
≥ pT3b	50 (26.6%)
Pathologic Gleason score	
7	81 (43.1%)
8	74 (39.4%)
≥ 9	33 (17.6%)
PSMs	
Overall	50 (26.6%)
In pT2 cancer	16 (8.5%)
In pT3 cancer	34 (18.1%)
Adjuvant treatment	
Overall	63 (33.5%)
Radiotherapy	25 (13.3%)
Hormonal therapy	38 (20.2%)
Salvage treatment	
Overall	35 (18.6%)
Radiotherapy	10 (5.3%)
Hormonal therapy	15 (8.0%)
Secondary after adjuvant therapy	10 (5.3%)
Follow-up duration, median (IQR), month	66.5 (13–94)
BCR	42 (22.3%)
Time to BCR, median (IQR)	10.5 (3.5–26)

**Fig. 2:**
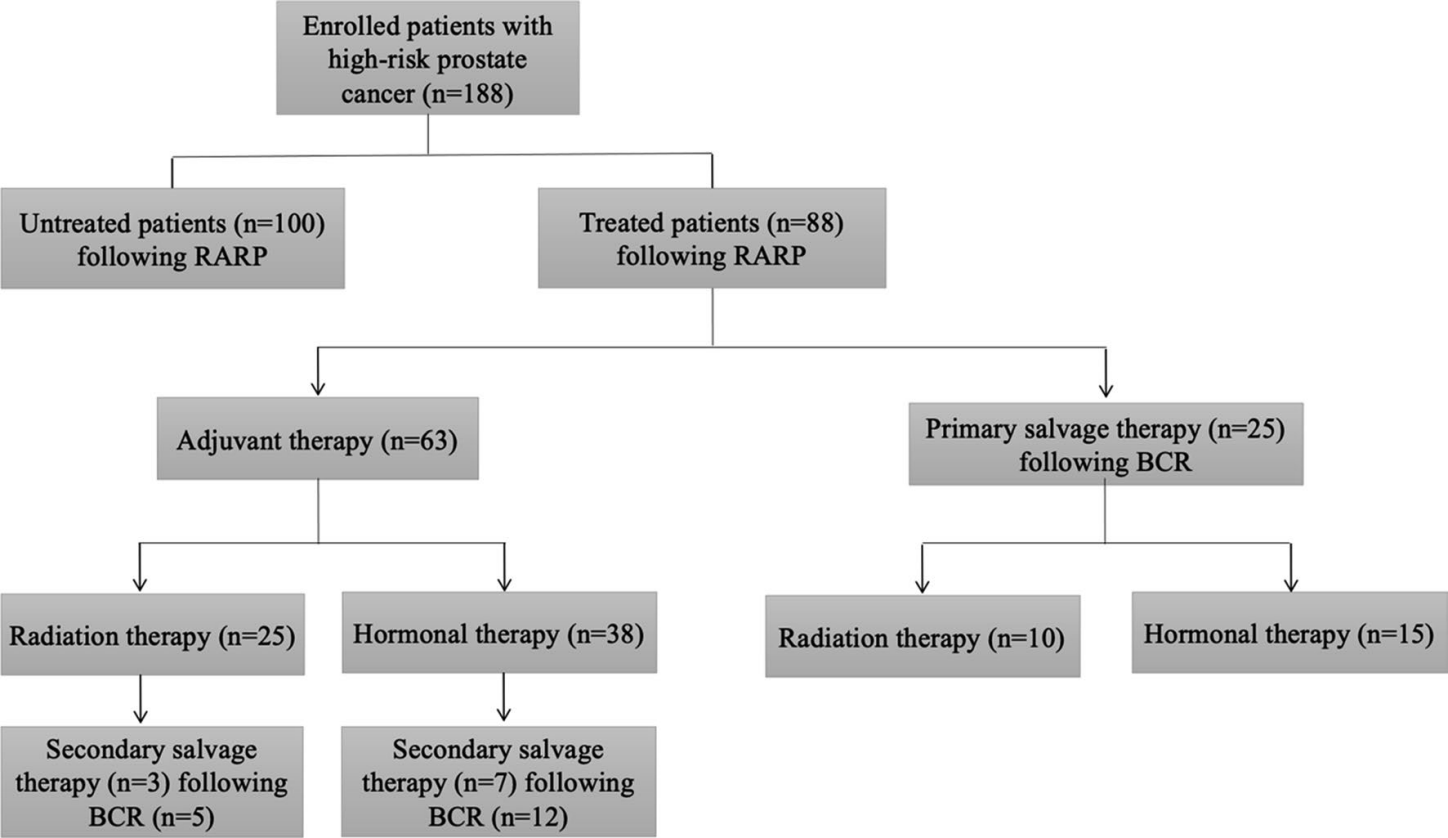
Treatment stratification tree based on the data of 188 patients with high-risk localized and locally advanced prostate cancer undergoing RARP

PSMs on postoperative pathologic examination were found in 50 of the 188 patients (26.6%). However, the rate of PSMs in patients with stage pT2 disease was lower (8.5%, 16 of the 188 patients). 63 of the 188 patients (33.5%) required adjuvant therapy. Adjuvant therapy with radiotherapy and hormonal therapy was utilized in 25 (13.3%) and 38 (20.2%) patients, respectively, whereas 35 of the 188 patients (18.6%) required salvage therapy. Primary salvage therapy after BCR was utilized in 10 patients with radiotherapy and 15 patients with hormonal therapy > 6 months following RARP. Additionally, secondary salvage therapy following primary adjuvant therapy was utilized in 10 patients with radiotherapy combination with hormonal therapy. We summarized the details of postoperative therapies administered following RARP (Fig. 2).

**Fig. 3:**
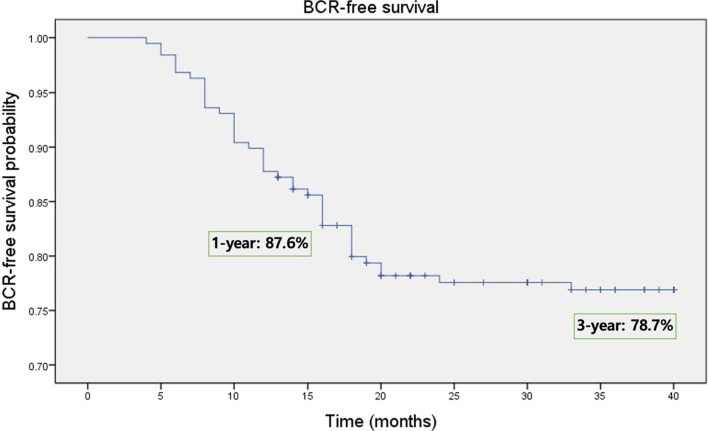
Kaplan–Meier curve showing estimated probability of BCR-free survival after RARP of 188 patients with clinically high-risk localized and locally advanced prostate cancer

The median follow-up duration was 66.5 (IQR, 13–94) months. BCR occurred in 42 patients (22.3%), and the median time to BCR was 10.5 (IQR, 3.5–26) months. The remaining 146 patients (77.7%) did not experience BCR during the follow-up period. BCR was achieved in 17 and 25 patients who received adjuvant therapy and salvage therapy, respectively. The median PSA level at the time of BCR was 0.5 (IQR 0.2–1.55) ng/mL. Figure 3 demonstrates the Kaplan–Meier estimates for BCR-free survival rates. The 1- and 3-year BCR-free survival rates were 87.6% (95% confidence interval [CI] 82.5%-92.4%) and 78.7% (95% CI 73.6%-87.3%), respectively. All patients with BCR underwent pelvic magnetic resonance imaging, bone scintigraphy, and chest and abdominal computed tomography. One patient was diagnosed with metastasis to the liver and brain at 24 months postoperatively. This high-risk locally advanced prostate cancer patient had a pre- and post-operative TNM stage of cT3aN0M0 and pT3bN1M0, respectively, with a Gleason score of 9. There were no cancer-related deaths. Of the 42 patients with BCR, 8 patients (19.1%) underwent observation, 15 patients (34.1%) were treated with hormonal therapy, 9 patients (21.4%) exhibited persistently elevated PSA levels without evidence of metastasis and were treated with salvage pelvic radiotherapy, and 10 patients (23.8%) following adjuvant therapy underwent secondary salvage radiotherapy and hormonal therapy. Finally, the 1-and 3-year CR-free survival rates were 97.5% (95% CI 91.5–98.8%) and 90.8% (95% CI 86.4–94.3%), respectively.

## Discussion

For patients with clinically localized prostate cancer and a life expectancy beyond 10 years, radical prostatectomy is the treatment of choice. Recently, more than 80% of radical prostatectomy procedures in the USA are performed with robotic assistance [23]. Recent literature reviews and meta-analyses show that RARP is associated with decreased rates of PSMs, improvements in potency and continence recovery at short-term follow-up, and shorter hospital stay compared to open and laparoscopic radical prostatectomy approaches in low- and intermediate-risk patients [24, 25].

However, 20–30% of patients continue to present with high-risk localized and locally advanced prostate cancer at the time of initial diagnosis [6–8]. Optimal treatment for those diagnosed with high-risk localized and locally advanced prostate cancer is controversial, and the role of radical prostatectomy for locally advanced high-risk prostate cancer remains a topic of debate due to several reasons, including discouragement of surgical management and evasion of RARP because of inexperience with the technique and potential difficulty in performing extended pelvic lymph node dissection [26].

Several series have recently reported the role of RARP in patients with high-risk localized and locally advanced prostate cancer. Despite its aggressive behavior, the prognosis of high-risk localized and locally advanced prostate cancer is not uniformly poor. In fact, high-risk prostate cancer is confined to the prostate in many patients, who may experience long-term progression-free survival after radical prostatectomy. Boylu et al. found that 15% of high-risk prostate cancer patients downgraded in the final pathological examination, and 77% of the high-risk patients did not need a secondary therapy (radiation or hormonal therapy) during the follow-up period [27]. Yossepowitch et al. analyzed radical prostatectomy outcomes in 957 patients with clinically localized high-risk prostate cancer and found that the cancer was confined to the prostate in 43% of the patients [28]. Compared with low- and intermediate-risk patients, there was a 3.3-fold increase in relapse hazard and higher likelihood of progression at five and ten years after radical prostatectomy. A multicenter study by Sooriakumaran et al. concluded that radical prostatectomy for patients with resectable distant metastases was safe in expert hands in the setting of meticulous patient selection [29]. Haese et al. already reported on 1,015 high-risk locally advanced prostate cancer patients (≥ pT3) treated with RARP and concluded that consequently, more than the surgical approach itself, the well-trained surgeon remains the most important factor to achieve satisfactory outcomes [5]. Gandaglia et al. found that RARP represents a well-standardized, safe, and oncological effective option in patients with locally advanced prostate cancer and pathologic stage, Gleason score, and PSMs should be considered to select patients for multimodal approaches [15]. Current EAU guidelines state that informing patients that no surgical approach (open-, laparoscopic- or robotic radical prostatectomy) has clearly shown superiority in terms of functional or oncologic results [30]. The adoption of radical prostatectomy as a treatment option for high-risk prostate cancer was based on the reported overall (and disease-specific) survivals rates of 87% (93%), 70% (83%), and 58% (71%) at 5, 10, and 15 years, respectively [31].

The big question remains whether radical prostatectomy is superior to radiotherapy combined with hormonal therapy. Several studies retrospectively compared radical prostatectomy with radiotherapy. Boorijian et al. retrospectively compared outcomes between radical prostatectomy and radiotherapy combined with hormonal therapy in patients with high-risk prostate cancer and found overall survival was significantly better in patients who underwent radical prostatectomy compared with patients who underwent external-beam radiotherapy with or without hormonal therapy; the authors also found that the risk of all-cause mortality was greater after radiotherapy with hormonal therapy compared to radical prostatectomy [32]. Zelefsky et al. found that cancer-free survival rates were comparable between radical prostatectomy and radiotherapy combined with hormonal therapy in patients with high-risk prostate cancer [33]. In that study, the absolute benefit of 7.8% in distant metastasis-free survival favored radical prostatectomy. Therefore, radical prostatectomy may be superior to radiotherapy combined with hormonal therapy in healthy patients with long life expectancy. Additionally, Boris et al. demonstrated the feasibility and durability of salvage RARP after failed radiotherapy and reported that the functional and oncologic outcomes of RARP were not inferior to those of open radical prostatectomy [34]. Salvage RARP may be another good option for the treatment of patients with organ-confined high-risk prostate cancer after failed radiotherapy.

In the present study, the median operative time was 185 min and the median intraoperative blood loss was 200 mL; only one patient with cardiovascular disease received intraoperative blood transfusion. These findings are not substantially different than those reported in a systematic review of RARP outcomes in patients with high-risk prostate cancer. Yuh et al. reported that the mean operative time was 168 min, the estimated blood loss was 189 mL, the mean length of hospital stay was 3.2 days, and the duration of catheterization was 7.8 days [6]. In the present study, the median duration of hospital stay and urinary catheter indwelling was 7 days due to nature of the Korean medical insurance. As on opinion of our institution, we present advantages of these long hospitalizations include the prevention of postoperative complications such as ileus and lymphocele.

In our study, the continence rates were 54.3%, 69.2%, 78.7%, and 88.3% at 1, 3, 6, and 12 months after RARP, respectively. The continence rates in the current study were consistent with the findings of Student et al. who demonstrated the benefits of early recovery of urinary continence in patients with low- or intermediate-risk prostate cancer undergoing RARP, and found similar continence rates (62.5%, 68.8%, 75.0%, and 86.7% at 1, 2, 6, and 12 months, respectively) [35]. We performed the detrusorrhaphy technique which is designed for thickening and strengthening the detrusor muscles from the posterior bladder neck to the bilateral dissected pedicles area; this technique is thought to prevent hyper-mobilization of the bladder neck area, thereby reducing stress urinary incontinence, and is considered to be important for continence recovery, as we have previously reported [17]. Furthermore, using a validated sexual function questionnaire, we found that the potency rate was 11.2% one month after RARP, which subsequently increased to 18.6%, 38.3%, and 56.4% at 3, 6, and 12 months after RARP, respectively. These outcomes are better than those reported by other studies using the same validated questionnaire in patients undergoing RARP. In their study examining nerve-sparing in salvage RARP, Bonet et al. reported that the 12-month potency rate was 25.6% in the good nerve-sparing group and that good nerve-sparing tended to be predictive of potency after salvage RARP [36]. In the current study, we performed athermal clipless intrafascial nerve-sparing technique if indicated (high-risk localized group); this technique might be associated with improved viable tissue preservation within the neurovascular bundles as we previously reported [17]. However, in high-risk locally advanced prostate cancer group, we dissected endopelvic fascia and performed wider dissection by clipping technique of neurovascular bundle due to guarantying postoperative oncologic outcomes.

In the present study, the rate of patients with stage pT2 organ-confined prostate cancer by postoperative pathologic assessment was 33.5%, similar to that reported in the systematic review of RARP-related outcomes by Yuh et al., who found that the average rate of organ-confined disease was 35% (range 7–48%) [6]. During the 3-year follow-up period in the present study, 100 of the 188 patients (53.2%) did not require secondary therapy such as radiotherapy or hormonal therapy. The rate of PSMs in the current study was 26.6%, in line with previous reports, as revealed in the systematic review by Yuh et al., in which the rate of PSMs was 35% (range 12–53%) [6]. The rate of PSMs was reduced to 8.5% in the patients with stage pT2 disease. During follow-up period, we perform mpMRI, chest and abdominal-pelvis computerized tomography scan, and bone scan for imaging in BCR if the PSA level is > 0.2 ng/mL. Prostate-specific membrane antigen (PSMA)-PET scan was substantially more likely to detect metastatic tumors in these men than the standard imaging approach used in many countries. Therefore, we will additionally perform PSMA-PET scan. In the present study, the median follow-up duration was 66.5 months, the median time to BCR was 10.5 months, and the overall BCR rate was 22.3% (42 of the 188 patients). Comparable to the present study findings, Kumar et al. reported that the overall BCR rate was 19.2% during a mean follow-up duration of 24.3 months and that the mean time to BCR was 7.9 months in patients with high-risk prostate cancer [37]. Our analyses revealing 3-year BCR-free and CR-free survival rates of 78.7% and 90.8%, respectively, are comparable to those reported in a study of patients with high-risk prostate cancer by Rogers et al. [38]. According to Jo et al. study, PSMs after RARP is one of the powerful predictors of BCR [39]. Based on these our results, we suggest that, when feasible, RARP should be considered in patients with high-risk localized and locally advanced prostate cancer if the patient accepts the surgical risk.

In patients with high-risk localized and locally advanced prostate cancer, RARP should be performed by skilled and experienced surgeons rather than beginners to reduce complications and to achieve optimal surgical results, given that radical prostatectomy is associated with high morbidity. A study by Punnen et al. provides further support for the effect of surgical experience on improved outcomes with RARP in patients with high-risk prostate cancer [2].

The limitations of the present study are its retrospective and noncomparative design, performance by only a single experienced surgeon in a single institution, and the small sample size. While the present study was not a randomized trial, we believe that the biases associated with the study design were minimal, given that the surgeon had already performed > 1000 RARPs between 2007 and 2020 and that the surgical methods in patients with high-risk localized and locally advanced prostate cancer are not challenging. Despite the ongoing follow-up of the study patients, our initial results suggest optimal functional and oncologic outcomes with RARP in patients with high-risk localized and locally advanced prostate cancer. Future studies should be conducted to include larger cohorts with longer follow-up periods to concomitantly compare the functional and oncologic outcomes of RARP with radiotherapy and/or hormonal therapy in patients with high-risk localized and locally advanced prostate cancer in a standardized fashion.

## Conclusions

RARP conferred long-term cancer control in most patients with high-risk localized and locally advanced prostate cancer and was a safe and feasible minimally invasive surgical alternative to radiotherapy or hormonal therapy in select patients. These results demonstrating the optimal functional and oncologic outcomes of RARP should be validated to assure the reproducibility of measurements in prospective randomized-controlled studies.

## Data Availability

The datasets used and analyzed during the current study are available from the corresponding author on reasonable request.

## Abbreviations

RARS: Robot-assisted radical prostatectomy
PSA: Prostate-specific antigen
PLND: Pelvic lymph node dissection
SHIM: Sexual Health Inventory for Men
IPSS: International Prostate Symptom Score
PSMs: Positive surgical margins
BCR: Biochemical recurrence
CR: Clinical recurrence
IQRs: Interquartile ranges
CI: Confidence interval
