# Correction to: Robot-assisted radical prostatectomy in the treatment of patients with clinically high-risk localized and locally advanced prostate cancer: single surgeons functional and oncologic outcomes

**DOI:** 10.1186/s12894-022-01033-4

**Published:** 2022-06-27

**Authors:** Tae Young Shin, Yong Seong Lee

**Affiliations:** 1 Department of Urology, Ewha Womans University Mokdong Hospital, Ewha Womans University College of Medicine, Seoul, 07985 Republic of Korea; 2 Department of Urology, Chung-Ang University Gwangmyeong Hospital, Chung-Ang University College of Medicine, Gwangmyeong, 14353 Republic of Korea; 3 Department of Urology, Hallym University Sacred Heart Hospital, Hallym University College of Medicine, Anyang, 14068 Republic of Korea; 4 Department of Urology, Chuncheon, Sacred Heart Hospital, Hallym University College of Medicine, Chuncheon, 24253 Republic of Korea

## Correction to: ﻿BMC Urology (2022) 22:49 https://doi.org/10.1186/s12894-022-00998-6

Following publication of the original article [1], the authors identified an error in affiliation information at the first page of the article. It has been corrected in this correction.

The original article [1] has been corrected

## References

[1] Shin, Lee (2022). Robot-assisted radical prostatectomy in the treatment of patients with clinically high-risk localized and locally advanced prostate cancer: single surgeons functional and oncologic outcomes. BMC Urol.

